# Mass Spectrometry Imaging of Specialized Metabolites for Predicting Lichen Fitness and Snail Foraging

**DOI:** 10.3390/plants9010070

**Published:** 2020-01-06

**Authors:** Alice Gadea, Mathieu Fanuel, Anne-Cécile Le Lamer, Joël Boustie, Hélène Rogniaux, Maryvonne Charrier, Françoise Lohézic-Le Devehat

**Affiliations:** 1 Univ Rennes, CNRS, ISCR (Institut des Sciences Chimiques de Rennes)—UMR 6226, F-35000 Rennes, France; alice.gadea@live.com (A.G.); joel.boustie@univ-rennes1.fr (J.B.); 2 Univ Rennes, CNRS, ECOBIO (Ecosystèmes, biodiversité, évolution)—UMR 6553, F-35000 Rennes, France; maryvonne.charrier@univ-rennes1.fr; 3 INRA, UR1268 Biopolymers Interactions Assemblies, F-44316 Nantes, France; mathieu.fanuel@inra.fr (M.F.); helene.rogniaux@inra.fr (H.R.); 4 UMR152 PharmaDev, Université de Toulouse, IRD, UPS, F-31400 Toulouse, France; anne-cecile.le-lamer@univ-tlse3.fr

**Keywords:** *Pseudocyphellaria crocata*, Lobariaceae, Chemical Ecology, Optimal Defense Theory, Mass Spectrometry Imaging, Lichens, Specialized Metabolites, *Notodiscus hookeri*

## Abstract

Lichens are slow-growing organisms supposed to synthetize specialized metabolites to protect themselves against diverse grazers. As predicted by the optimal defense theory (ODT), lichens are expected to invest specialized metabolites in higher levels in reproductive tissues compared to thallus. We investigated whether Laser Desorption Ionization coupled to Mass Spectrometry Imaging (LDI-MSI) could be a relevant tool for chemical ecology issues such as ODT. In the present study, this method was applied to cross-sections of thalli and reproductive tissues of the lichen *Pseudocyphellaria crocata*. Spatial mapping revealed phenolic families of metabolites. A quantification of these metabolites was carried out in addition to spatial imaging. By this method, accumulation of specialized metabolites was observed in both reproductive parts (apothecia and soralia) of *P. crocata*, but their nature depended on the lichen organs: apothecia concentrated norstictic acid, tenuiorin, and pulvinic acid derivatives, whereas soralia mainly contained tenuiorin and pulvinic acid. Stictic acid, tenuiorin and calycin, tested in no-choices feeding experiments, were deterrent for *N. hookeri* while entire thalli were consumed by the snail. To improve better knowledge in relationships between grazed and grazing organisms, LDI-MSI appears to be a complementary tool in ecological studies

## 1. Introduction

Lichens are symbiotic organisms that use sexual and vegetative reproductions. As lichens are slow-growing organisms, integrity of sexual structures (apothecia) and vegetative propagules (soredia, isidia) should be crucial to lichen fitness. 

Localization of specialized also called secondary metabolites in an organism depends on their functional roles [1,2,3,4]. The optimal defense theory (ODT) defined by Mc Key postulates that specialized metabolites are primarily allocated to organism parts of high fitness value, high risk of predation, or both [5,6]. For example, young leaves are considered more valuable than old leaves and often possess the greatest concentrations of specialized metabolites [7]. Similarly, reproductive parts, as flowers and fruits, are more difficult to replace than vegetative parts and frequently possess high levels of resistance to herbivores [8]. In lichen, specialized metabolites are involved in many functions that influence lichen ecology as sun screening protection, allelopathy, antioxidant, antimicrobial activities or protection against lichen grazers [9], which may also influence their distribution in the lichen thalli. The concept of ODT in lichens was first evocated by Hyvärinen and al., studying in three foliose lichens the intrathalline variation of specialized metabolites between reproductive parts and vegetative ones [10]. The authors highlight that amounts of specialized metabolites in the studied species are higher in the reproductive parts, independently of the type of dispersal and the biochemical origin of the metabolites. These specialized metabolites are mainly phenolic compounds belonging to depsides, depsidones, dibenzofuranes, or pulvinic acid derivatives [11,12]. 

Most of the publications consider metabolite localization in the thallus using spot tests (reagents directly applied on the lichen thallus) or after extraction and analyses of specific parts of the lichen, without approaching the fine-scale spatial distribution of metabolites inside the lichen thallus. A more precise distribution of metabolites could be obtained by in situ imaging techniques that artificially divide the tissue into pixels whose dimensions define the resolution of the image. For example, classical fluorescent techniques are based on the molecule chromophore, where metabolites are only differentiated with their emission wavelength (i.e. flavonoids and terpenoids at 525 nm approximately), so it appears to be a nonselective targeting [13,14,15]. More recently, Mass Spectrometry Imaging (MSI) has become a powerful tool to assess the spatial distribution of metabolites in organisms [16,17,18,19,20,21]. The main advantages of MSI are in situ analyses, bypassing solubility issue encountered in mass spectroscopy in solution and avoiding the sample degradation. Laser Desorption ionization–Mass Spectrometry Imaging (LDI-MSI) has previously been applied on lichen cross-sections, which allowed discussing the roles of some metabolites in chemical ecology [20,21]. As reported previously [20], the depside miriquidic acid was isolated for the first time in the strongly competitive lichen *Ophioparma ventosa*. This compound was imaged by LDI-MSI and it was located in the basal medulla of the lichen thallus. This appears to be compatible with the fact that this compound could come initially from a Miriquida species known to be parasitized by this over-growing species [20]. Similarly, in situ imaging by mass spectrometry of primary and specialized metabolites from the lichen *Usnea taylorii* demonstrated that primary metabolites such as arabitol stimulates the snail in its food choices [21].

In the present study, we applied the chemical ecology concept of ODT on a case study, the lichen-snail trophic interaction between the lichen *Pseudocyphellaria crocata* and the snail *Notodiscus hookeri* respectively. The foliose lichen *Pseudocyphellaria crocata (L.)* Vain. (Lobariaceae) was chosen because sexual and asexual reproductive structures were clearly separated in the thallus and because of its high chemical diversity with depsidones (stictic acid derivatives), depsides (tenuiorin and derivatives), and pulvinic acid derivatives [22,23,24,25,26]. Spatial mapping of specialized metabolites in *P. crocata* was performed by LDI-MSI on sections of the reproductive and vegetative parts of lichen thalli. The lichenophagous snail *Notodiscus hookeri* was used. This terrestrial snail lives in subantarctic islands and feeds only on lichens [27,28]. Previous studies have shown that the feeding strategy of *N. hookeri* is based on searching for lichen parts with attractive primary metabolites like polyols, even when such nutrients are mixed with potentially deterrent metabolites [21,29]. Therefore, snail gustatory responses are governed by nutrient availability [21]. After consumption of *P. crocata,* the fate of lichen metabolites in the snail gut has been followed [22]. Tridepsides and pulvinic acid derivatives were excreted, whereas stictic acid and its derivatives could not be identified in any part of the snail gut nor in feces [22].

To know whether spatial distribution of specialized metabolites could serve as a proxy for predicting lichen defense, i.e. for evaluating the risk of predation by lichen grazers, we addressed two aims: (i) Identifying and mapping the molecules allocated to the reproductive parts of the lichen by LDI-MSI. (ii) Investigating whether snail gustatory responses match well with MSI of the metabolites. According to the work in [22], we expected stictic acid to be more deterrent to the snail than tenuiorin or calycin, suggesting stictic acid to be more abundant in reproductive parts of the lichen. This assumption was tested in no-choice feeding experiments where each of the metabolites, embedded in starch gel, was offered to adult snails at a concentration similar to that found in *P. crocata*. 

## 2. Results

### 2.1. Morphological and Chemical Characterization of the Lichen Pseudocyphellaria crocata and Spatial Mapping of Metabolites by LDI-MSI 

**Figure 1:**
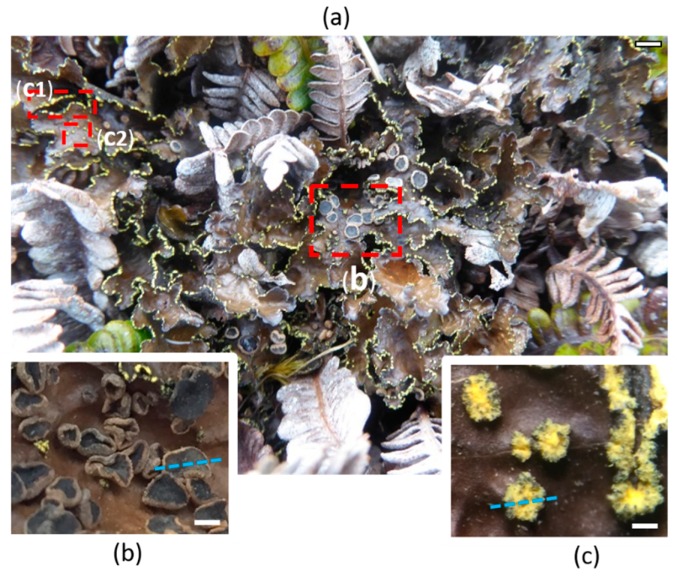
Reproductive parts of the lichen *P. crocata*. The thalli of the lichen species collected on the fern *Blechnum penna-marina* (**a**). Insets correspond to the sexual reproductive parts apothecia (**b**) and asexual reproductive part soralia (**c**) which are marginal (**c1**) and laminal (**c2**). Blue lines allow visualization of the cross sections for MSI experiments. Scales bars correspond to 1 cm, 500 µm, and 100 µm in panels (a–c), respectively.

The foliose lichen *P. crocata* has a large brown thallus (more than 10 cm diameter) with both apothecia and soralia (Figure 1). Unlike specimens from New Zealand or Ecuador, Crozet islands specimens of *P. crocata* get numerous small brown-red lecanorine apothecia (1–3 mm) [30,31,32]. Yellow soralia, which are both marginal and laminal, are delimited structures containing small powdery granules named soredia that consists in photobiont cells surrounded by fungal hyphae.

**Figure 2:**
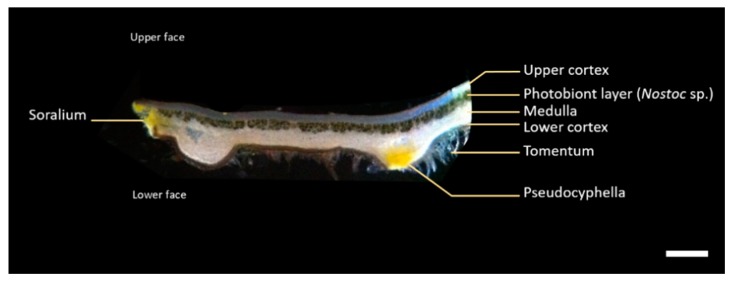
*P. crocata* thallus cross section that shows four distinctive layers and the basal part fixed to the substrate, called tomentum. Scale bar corresponds to 500 µm.

Thallus cross-section shows a heteromerous structure with four layers of interlaced hyphae: the brown upper cortex, formed by densely agglutinated fungal hyphae; the blue-green photobiont (*Nostoc* sp.) layer where algal cells are embedded in rather densely interwoven fungal hyphae; the white medulla made by loosely interwoven fungal hyphae and the brown lower cortex bearing tomentum that allow the lichen fixation on its substrate. Yellow pseudocyphellae appear as pores on the lower cortex, where medullary hyphae extend to the lower surface (Figure 2). 

**Figure 3:**
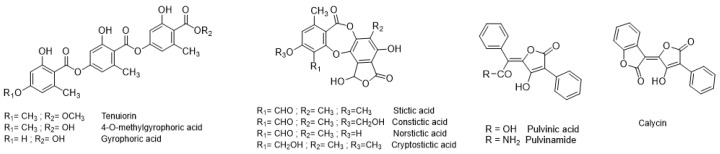
Structures of the phenolic specialized metabolites identified in *P. crocata.*

Preliminary LDI-MS analyses of an acetone extract of the whole thalli of *P. crocata* were performed to defined the best ionization conditions for the further LDI-MS imaging experiments. The results confirmed the presence of three family scaffolds: depsides (tenuiorin derivatives), depsidones (stictic acid derivatives) and pulvinic acids derivatives (Figure 3, Table 1). These analyses revealed that pulvinic derivatives were the best ionized metabolites (Figure S1). Deprotonated calycin at *m*/*z* 305 [M-H]^−^ was observed as well as characteristic fragments of pulvinic derivatives at *m*/*z* 263 and 233 [33]. Tridepsides are unstable compounds that undergo rapid fragmentation in mass spectrometry. This general trend was confirmed as no deprotonated molecule of tridepsides (tenuiorin, gyrophoric acid and 4-O-methylgyrophoric acid) was detected. However, some specific fragments could be observed, particularly fragment with *m*/*z* 149 corresponding to the loss of two aromatic units after acyl cleavages. Depsidones (stictic acid and derivatives) were detected, but with low relative intensities at *m*/*z* 357, 371, 385, 387 and 401 (Figure S1).

**Figure 4:**
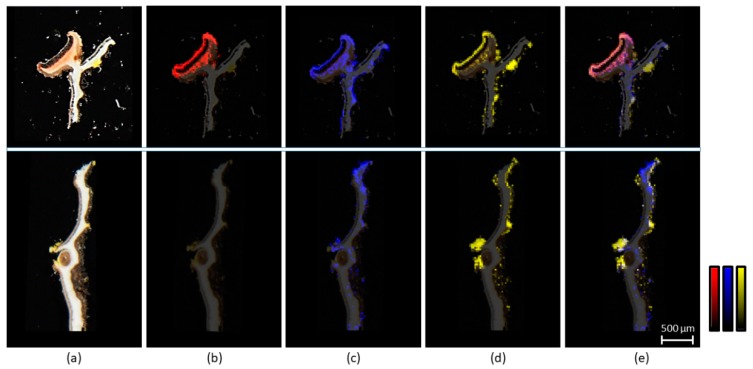
Distribution of the specialized metabolites in the lichen *P. crocata*. Optical image (**a**) of the cross-sections of an apothecium (upper panel) and a thallus with soralium (lower panel). Mass spectrometry imaging shows (**b**) norstictic acid at *m*/*z* 371, (**c**) fragment of depsides at *m*/*z* 149 and (**d**) calycin at *m*/*z* 305. Image (**e**) compiled all the previous results. Intensity scales were adjusted to maximize the visualization of each family compound.

In situ LDI-MSI experiments were performed on two types of samples of *P. crocata,* slices of thallus with apothecia or with soralia (Figure 4a). A sectorial distribution of the metabolites in the lichen was observed. No depsidone or depsidones fragments were detected in the vegetative thalli. Norstictic acid at *m*/*z* 371 was the only depsidone detected by LDI-MSI in the apothecia (Figure 4b). Fragments of depsides at *m*/*z* 149 were present in the medulla but also accumulated in apothecia and soralia (Figure 4c). Pulvinic acid derivatives and particularly the major calycin are yellow-orange metabolites, which were logically encountered in the yellow soralia of the lichen and on pseudocyphellae. Most interestingly, these metabolites were observed also in apothecia (Figure 4d). It is noteworthy that characteristic fragments of pulvinic derivative at *m*/*z* 263 and *m*/*z* 233 have the same localization as calycin pseudomolecular ion at *m*/*z* 305. 

### 2.2. Quantification of Lichen Metabolites in P. crocata by HPLC-DAD-MS ESI(−)

Quantification by High-Performance Liquid Chromatography–Diode Array Detector (HPLC-DAD) of the specialized metabolites in the acetone extracts of thalli and apothecia was summarized in Table 1. Tenuiorin was the main metabolite in the acetone extract of the thalli. In the acetone extract of the apothecia, norstictic acid was the major compound and it was twenty times more concentrated than in thallus. Calycin was in higher concentrations in the acetone extract of the thallus (containing soralia) than in apothecia. Acetone extract of soralia enriched thalli confirmed high concentrations of calycin and presence of other pulvinic acid derivatives (data not shown). The physicochemical properties such as theoretical partition coefficient (logP) and water solubility may influence the localization of the metabolites in the lichen. We used ALOGPS 2.1 freeware to estimate the theoretical partition coefficient and the water solubility. Depsides were less soluble in water than depsidones and pulvinic acids. The depsidone norstictic acid was more hydrophilic than stictic acid, which is in accordance with the phenolic substituents (Figure 3).

### 2.3. No-Choice Feeding Experiments with N. hookeri

**Figure 5:**
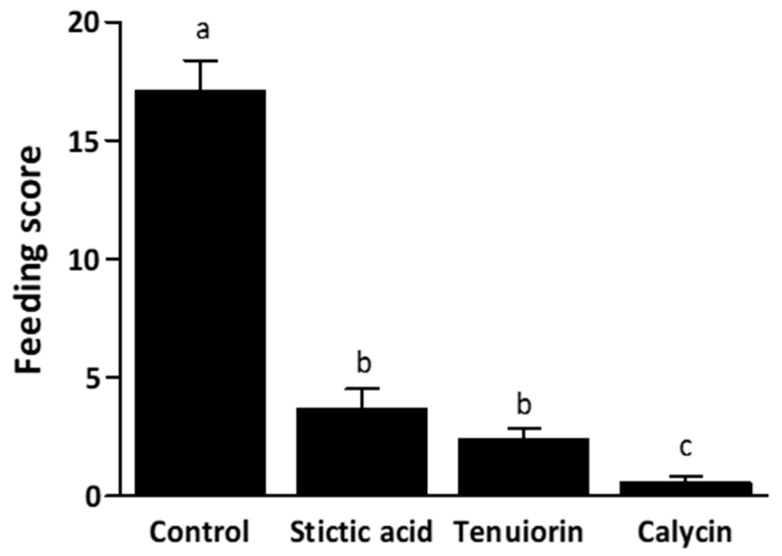
Gel consumption, estimated by a feeding score (Mean±s. e.), by the snails (N = 150 × four groups), according to the metabolite tested on starch gel. Starch gel without metabolites was considered as the positive control. Significant differences between positive control and metabolite containing gels (stictic acid, tenuiorin, or calycin) are highlighted by the lowercase superscript letters a, b and c.

The tested metabolites—calycin, stictic acid, and tenuiorin—were added separately to starch gels at the mean concentrations found in the apothecia (Table 1). After 48h, gel-mixed metabolites had a significant impact on their consumption by snails (Chi^2^ = 119.91, df = 3, *p* < 0.001; Figure 5). Gel consumption by snails was strongly dependent on its chemical composition, the three specialized metabolites being deterrent compared to the control. Gels containing calycin were significantly less consumed than those containing stictic acid or tenuiorin (Tukey tests, *p* < 0.001). In contrast, tenuiorin and stictic acid were equally eaten (Tukey test, *p* = 0.508). 

## 3. Discussion

Mass spectrometry imaging is an innovative technique that started to develop more than twenty years ago, and was first used for imaging proteins and peptides particularly in medicine [34]. More recently, this technique was applied for the spatial mapping of small molecules in several areas at the interface between biology and chemistry [35]. In our work, LDI-MSI proved to be a relevant method to localize lichen specialized metabolites in a chemical ecology context, allowing a precise location of the metabolites inside the thallus especially when it was not possible to separate some lichens parts (i.e., soralia from the rest of the thallus). 

Tenuiorin, the main specialized metabolite of *P. crocata* was previously described with a cortical localization in *Pseudocyphellaria* species [36]. LDI-MSI results are in opposition with this previous statement based on thalline reactions. Indeed, tenuiorin is a medullary metabolite in the lichen *P. crocata*. Some unexpected results were highlighted with depsidones detected by LDI-MSI. In the previous LC-DAD-MS ESI(-) profiling of *P. crocata*, stictic acid was detected and quantified as the main depsidone in the acetone extract of the entire lichen thalli [22]. This metabolite was also detected by LDI-MS in the acetone extract of whole thalli of *P. crocata*. Based upon the literature, we expected to observe depsidones and particularly stictic acid in the lichen medulla [36,37,38]. Surprisingly, stictic acid was not detected by LDI-MSI both in the vegetative thalli and in reproductive parts of *P. crocata*. We could explain the absence of ionization of stictic acid in LDI-MSI by the low metabolite concentrations in the lichen matrix compared to the acetone extract. Indeed, fungal hyphae are a mixture of chitin and polysaccharides, which may limit some specialized metabolites desorption by the laser. The screening by LDI-MSI of the lichen cross-sections also revealed that norstictic acid was confined to the apothecia of the lichen. This metabolite accumulated 20 times more in apothecia than in the rest of the lichen thalli. We speculate that the higher water solubility of norstictic acid compared to stictic acid might be involved in protecting the reproductive structure during intense rainy episode by leaching at the surface of apothecia, as it is known for polyols [39]. Pulvinic acid derivatives show different sectorial localizations according to the lichen species, being for instance cortical in the lichen *Letharia gracilis* [40], medullar in *Pseudocyphellaria aurata* [41], whereas in *P. crocata*, pulvinic acid derivatives are clearly restricted to soralia and pseudocyphellae. 

Unlike in plants, lichen chemical defenses are not induced by phytophages’ attacks and are most likely constitutive [42]. The presence of all families of metabolites in the apothecia suggests that sexual reproductive parts in *P. crocata* are highly protected against lichenophages. Various examples of an accumulation of specialized metabolites in parts of lichens with high fitness value as reproductive parts were described, including parietin, physodic acid, atranorin, pinastric acid, *m*-scrobiculin, and cytochalasin [10,43,44,45]. However, accumulation of metabolites in reproductive parts is not the rule for all metabolites. The dibenzofurane usnic acid is equally distributed into the external layers of the entire lichen *Usnea taylorii* (both branches and apothecia) and the lichenivore *N. hookeri* must overcome the deterrent effect of this metabolite to gain the polyol, arabitol [21]. Furthermore, accumulation of specialized metabolites might not only protect the lichen apothecia from lichenivores grazing but also from other external stress as UV radiation or oxidative stress [9,46]. One can assume that metabolites as calycin in the apothecia acted as UV filter to protect spores during their maturation within asci, avoiding a potential DNA degradation of the spores [9]. 

No-choice feeding experiments highlighted the deterrent effect of tenuiorin, stictic acid and calycin for *N. hookeri* and the latter had stronger repellence despite it was the less concentrated in the lichen. These three specialized metabolites were yet known to play a role in defense against predation by gastropods. For example, lichens containing stictic acid were significantly more eaten by the snail *Cepaea hortensis* after removal of the metabolite [47], and the same snail species was observed avoiding soralia of *P. crocata* [48]. In the same way, stictic acid-rich chemotype of *Lobaria pulmonaria* was avoided by gastropods while stictic acid-free chemotype was grazed [49]. Pulvinic acid derivatives (including calycin) are well known powerful anti-lichenophages [48,50] and their detection in the reproductive parts of *P. crocata* highlights an optimal defense strategy for soralia [10,43]. In contrast to other snails [45], *N. hookeri* was able to eat calycin-containing soralia because their characteristic yellow pigment was retrieved in the snail feces, although calycin was the highest repellent compound in starch gels. Conversely, stictic acid was absent from the digestive tract and feces of the snail *N. hookeri* [22], but it was sparsely consumed in starch gels. A seemingly contradictory result emerged between what we expected to happen during feeding, rejection of the depsidone, and what the snail actually did, consuming gels with stictic acid more than gels with calycin. Therefore, *N. hookeri* does not behave similarly when fed on lichens or on starch gels mixed with metabolites [27]. Yet, previous studies that focused on snail grazing on *Argopsis friesiana* or *Usnea taylorii* highlighted the phagostimulant role of primary metabolites that may overcome the deterrence of specialized metabolites [20,27]. In *P. crocata*, primary metabolites were not quantified in the different lichen parts, but arabitol was the main carbohydrate found in the entire lichen with a mean value (±s.e.) of 58.15 ± 2.38 mg∙g^−1^ DM (data not shown). Among depsidones, norstictic acid was not tested in the present study (See §4.4), but since it was by far more concentrated than stictic acid in apothecia of *P. crocata*, it should deter more efficiently grazing. It might be suggested that apothecia of *P. crocata* would be avoided because of their richness in depsidones combined with poorness in arabitol, whereas arabitol richness in the soralia close to thallus would counteract the deterrence of depsides and pulvinic acid derivatives. Specialized metabolites present in the reproductive parts, as soralia, might be stimulant or repellent to snail grazing depending on phytophagous species ability to circumvent the toxicity associated with any of these substances. Previously, Gadea et al [21] recorded a reduced excretion of tenuiorin in the snail feces and suggested that the conversion of tenuiorin in 4-*O*-methylgyrophoric acid in the digestive tract should circumvent tenuiorin toxicity.

## 4. Materials and Methods 

### 4.1. Biological Materials 

Lichen *Pseudocyphellaria crocata (L.)* Vain. (Lobariaceae) [51] was harvested on Possession Island, Crozet Islands, during 2015 Austral summer, in grassy coastline dominated by Poaceae (*Poa cookii*, *Agrostis magellanica*) and fernbrakes (*Blechnum penna-marina*) of Pointe Basse (46°22’5.48” S; 51°43’26.39” E, 100 m). Voucher specimen was deposited at the herbarium of the faculty of Pharmacy of Rennes 1, Department of Pharmacognosy and Mycology, under the reference REN000148. 

Isolated metabolites (tenuiorin (JB/A/195), stictic acid (JB/A/141) and calycin (JB/A/185)) used for calibration and snails’ behavior experiments belongs to the library of pure lichen compounds of UMR 6226, isolated during previous phytochemical investigation.

Adult individuals of *Notodiscus hookeri* Reeve (Charopidae) were collected on Possession Island, Crozet Islands, during 2015 Austral summer. 

### 4.2. Mass Spectrometry Imaging

Samples preparation and data acquisition were performed under the same conditions as previously described by Gadea et al. [21]. Briefly, *Pseudocyphelaria crocata* samples were hand-cut using a razor blade to afford slices approximately 100 μm thick. Lichen slices were fixed on a carbon-conductive adhesive tape that was, itself, fixed on an indium tin oxide (ITO) slide (Bruker Daltonics, Bremen, Germany, cat. no. 237001). All MSI measurements were performed using an Autoflex-Speed MALDI-TOF/TOF spectrometer (Bruker Daltonics, Bremen, Germany) equipped with a Smartbeam laser (355 nm, 1000 Hz) and controlled using the Flex Control 3.4 software package. The mass spectrometer was used in the reflectron mode with a negative polarity. Spectra were acquired in the mass range of *m*/*z* 100 to 1000 for all (x, y) coordinates corresponding to the imaged tissue. The laser raster size was set at microns. The signal was initially optimized by manually adjusting the laser power and the number of laser shots fired. Accordingly, full-scan MS experiments were run by accumulating 400 laser shots per raster position and by using the laser power leading to the best signal-to-noise ratio. Image acquisition was performed using the Flex Imaging 4.0 (Bruker Daltonics) software package. 

### 4.3. HPLC-DAD-MS Analysis and Prediction of LogP and Water Solubility of Specialized Metabolites

Apothecia were carefully separated from vegetative thalli of *P. crocata*. Both apothecia (n = 3) and vegetative thalli (n = 3) samples were grounded separately with mortar and pestle in liquid nitrogen (m = 100 mg). Each sample was extracted with 100% acetone for 20 minutes (3 × 500 μL, at room temperature). Acetone extracts were profiled by Liquid Chromatography–Diode Array Detector (LC-DAD) (Shimadzu®, Marne La Vallée, France) and a mass spectrometer (MS) (Advion® expression CMS, Ithaca, USA) according to Gadea et al. [29]. The spectral data from the photodiode array detector were collected 48 min over the 200 to 500 nm range of the absorption spectrum and the chromatograms were plotted at the maximum wavelength of absorption (λmax) of main metabolites. Metabolites were identified according to the retention time and UV spectra as well as based on mass spectra for both the standards and the samples under the same chromatographic method [22]. Concentrations were obtained on the basis of calibration curves and were expressed in mg∙g^−1^ DM of the lichen part. Procedure for the validation of the metabolite quantification is described in supporting materials. LogP and water solubility were predicted with ALOGPS 2.1 online software (VCCLAB, Lausanne, Swiss), on the basis of molecule’s smiles [52]. 

### 4.4. Snails No Choice Experiments on Isolated Compounds

Six hundred adult snails were separated in four subgroups: one control and three tested groups of 150 snails each (tenuiorin, stictic acid, and calycin). Stictic acid was preferred to norstictic acid because of it availability in the laboratory and because it was the main depsidone quantified in the entire lichen in a previous work [22]. Compounds making crystals were dissolved in acetone before being mixed in the starch gels. 

No-choice experiments were performed following the methodology of Gadea et al. [21]. Metabolites (tenuiorin, stictic acid, and calycin) were added to the gel at the same concentrations as those quantified in the apothecia (22 mg∙g^−1^, 4 mg∙g^−1^, and 0.5 mg∙g^−1^, respectively). After 48 hours, gel consumption was evaluated by a feeding score. The score was calculated with a calibrated grid placed on the gel pictured that provided the area consumed, and a correction factor was used depending on the thickness of gel consumed [20].

### 4.5. Statistical Analyses

In the no-choice experiments, to determine if metabolites were differently consumed compared to the control, a likelihood ratio test was used on a generalized linear model (GLM, distribution: negative binomial, link function: log). Tukey’s post hoc pairwise comparisons were realized. Statistical analyses were made using R software V. 3.4.3 [53]. The R packages used were car, emmeans, lme4, MASS, and RVAideMemoire.

## 5. Conclusions

LDI-MSI appeared to be a complementary tool for lichen-lichenivores interactions studies. This technique could also be interesting with others chemical ecology issues as photoprotection, allelopathy or antimicrobial activities. Most of the specialized metabolites were observed in the reproductive tissues of the lichen *Pseudocyphellaria crocata*. However, to monitor their distribution by LDI-MSI, metabolites must absorb at 355 nm, which is the laser wavelength of the instrument. Therefore, metabolites that do not contain aromatic rings cannot be detected by this method. This detection technique is direct, precise and sensitive, but since the observed signal intensity depends on the physicochemical properties of the molecule and on the molecular complexity of the tissue, it is not a quantitative measurement. Therefore, LDI-MSI should be complemented by a targeted quantification of the metabolites in the different lichen tissues using for example laser microdissection. To assess the ODT theory, LDI-MSI needs to be performed on both primary and specialized metabolites when used in ecological studies focusing on plant-eating invertebrates.

## Figures and Tables

**Table 1:** Main phenolic specialized metabolites families: quantification in the acetone extracts of thalli, soralia, and apothecia, including physicochemical properties and fragmentation in negative mode.

	Specialized Metabolites Described in *P. crocata*	Molecular Mass (g/mol)	LogP ^1^	Water Solubility (× 10^4^)	Levels of Specialized Metabolites in Thalli ^2^ (mg∙g^−1^ DM)	Levels of Specialized Metabolites in Apothecia ^2^ (mg∙g^−1^ DM)	Main MS Fragments *m*/*z* Expected in Negative Mode ^3^
**Depsides**	Tenuiorin	496.471	4.26	0.10	56.03 ± 9.98	21.90 ± 1.48	**149**,167, 313, 331, 495
	Gyrophoric acid	468.417	3.79	0.17	2.19 ± 0.31	3.23 ± 0.47	**149**, 167, 317, 467
	4-O-methylgyrophoric acid	482.441	4.10	0.17	5.85 ± 1.13	6.62 ± 0.65	**149**, 167, 299, 331, 449, 481
**Depsidones**	Stictic acid	386.312	1.67	3.46	3.84 ± 0.70	4.23 ± 0.35	285, 341, 385
	Norstictic acid	372.285	1.64	6.91	2.96 ± 1.06	64.01 ± 11.68	327, **371**
	Constictic acid	402.311	0.97	3.98	2.75 ± 0.46	2.14 ± 0.14	357, 401
	Cryptostictic acid	388.328	1.10	6.91	0.60 ± 0.07	1.07 ± 0.18	343, 387
**Pulvinic acid derivatives**	Calycin	306,274	2.67	2.08	1.62 ± 0.20	0.51 ± 0.03	233, **305**
	Pulvinic acid	308.289	3.03	2.08	0.41 ± 0.06	0.35 ± 0.02	**263**, 307
	Pulvinamide	307.305	2.22	1.44	0.22 ± 0.02	0.18 ± 0.02	**263**, 306

^1^ theoretical partition coefficient; ^2^ Mean ± s.e, n = 3 samples; ^3^ Observed *m*/*z* in LDI-MSI are in bold.

## Supplementary Materials

The following are available online at https://www.mdpi.com/2223-7747/9/1/70/s1, Text S1: Experimental quantification and validation, Table S1: Results of various parameters of validation studies for tenuiorin, stictic acid and calycin quantification, Figure S1: LDI-MS spectrum (negative ionization mode) of the acetone extract of *Pseudocyphellaria crocata*.
