# Supplementary material for: Mass Spectrometry Imaging of Specialized Metabolites for Predicting Lichen Fitness and Snail Foraging
Source: Plants (Basel). 2020 Jan 6;9(1):70. doi: 10.3390/plants9010070 (PMC7020473; doi:10.3390/plants9010070)
Supplement: Supplementary file 1 [file plants-09-00070-s001.pdf]

plants MDPI

Supplementary materials

Mass Spectrometry Imaging of specialized metabolites for predicting lichen fitness and snail foraging.

Text S1: Experimental quantification and validation

All validation parameters were determined following the International Conference on Harmonization (ICH) Guidelines (ICH, 2005) using HPLC analyses, and the following characteristics were evaluated: linearity, limits of detection (LOD) and quantification (LOQ), repeatability inter-day and intra-day. Four stock standard solutions of each metabolites were prepared by dissolving approximately 2 mg in 4 mL tetrahydrofuran to reach a concentration of 0.5 mg mL^{-1}. Then, six working standard solutions of each metabolite were prepared by appropriate dilution of each stock solution with acetonitrile to generate concentrations at the appropriate ranging (see Table S1) for the external standard calibration curve and the determination of the regression line. Hence, the concentrations of tenuiorin, stictic acid and calycin in *P. crocata* were calculated, based on peak areas. The limits of detection and quantification were determined from the y-intercept standard deviation and the slope of the calibration curve. For the calculation of the intra-day repeatability, a dilution of each metabolite was injected five times the same day. These assays were repeated on three different days for inter-day repeatability. The coefficient of variation and standard deviation were then calculated. Coefficients of variation of less than 10% for intra-day and for inter-day were accepted. The results of the validation are available in Table S1.

Table S1 : Results of various parameters of validation studies for tenuiorin, stictic acid and calycin quantification

Parameters	Tenuiorin	Stictic acid	Calycin
Lambda (nm)	254	254	365
Calibration curve equation	y = 50583626x - 1214979	y = 82631492x - 565837	y = 54078856x - 61714
Correlation coefficient value (R^2)	0.973	0.9288	0.984
LOD (µg.mL^{-1})	7.211	3.669	0.546
LOQ (µg.mL^{-1})	32.010	28.208	4.484
Calibration range (µg.mL-1)	25.00 - 200.00	7.81 - 125.00	1.93 - 31.25
Intraday precision (RSD, n=5)	6.3 %	less than 2%	6.8 %
Interday precision (RSD, n=3)	6.3 %	less than 2%	8.4 %

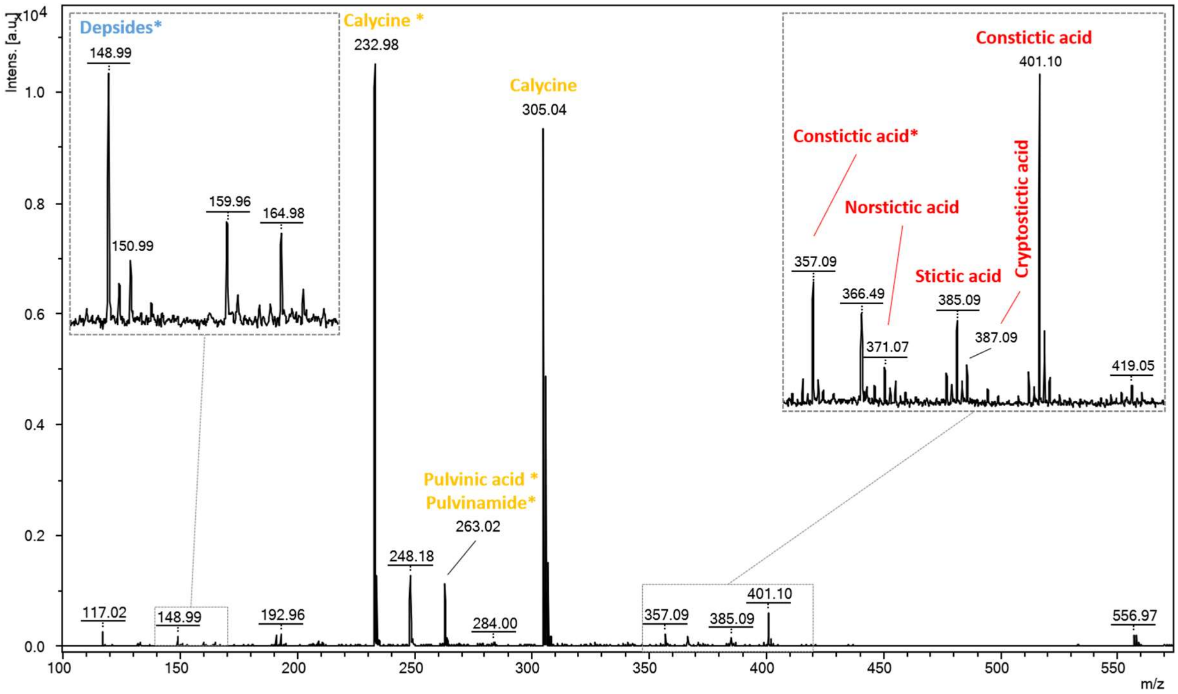

Figure S1. LDI-MS spectrum (negative ionization mode) of the acetone extract of *Pseudocyphellaria crocata.* Depside fragments are in blue, depsidones (ions and fragments) in red and pulvinic acid derivatives in yellow. The * designed common fragments of each families of molecules. For LDI-MS measurement, 1 mL of the acetone extract was deposited on a polished steel MALDI target plate at a concentration of 10 mg.mL-1 and analyzed in negative ionization mode
